# Nutritional Status, Dietary Habits, and Physical Activity in Older Adults from Manta, Manabí

**DOI:** 10.3390/foods11233901

**Published:** 2022-12-03

**Authors:** Arencibia Moreno Ricardo, Hernández Gallardo Damaris, Linares Girela Daniel, Linares Manrique Marta

**Affiliations:** 1 Facultad de Ciencias de la Salud, Universidad Técnica de Manabí, Portoviejo 130105, Ecuador; 2 Facultad de Ciencias de la Educación, Universidad Laica Eloy Alfaro de Manabí, Manta 130214, Ecuador; 3 Laboratorio de Antropología Física, Universidad de Granada, 18071 Granada, Spain; 4 Facultad de Ciencias de la Salud de Melilla, Universidad de Granada, 52071 Melilla, Spain

**Keywords:** older adults, dietary habits, nutritional status, physical activity, healthy eating index

## Abstract

Defining the nutritional status and physical activity level of older adults makes it possible to guide them toward healthy lifestyles. Objective: The aim of this study was to evaluate dietary habits, nutritional status, and physical activity engagement in older adults living in the city of Manta, Manabí. Methods: An observational, descriptive, and cross-sectional study of 130 older adults was conducted to determine nutritional status via anthropometry, self-reported frequency of the consumption of foodstuffs, calculation of the healthy eating index (IAS), and physical activity patterns. Results: Average age was 71.62 ± 4.34 years, whilst 83.07% of participants were at nutritional risk due to being overweight or obese. Dietary habits in males were characterized by the consumption of three meals a day, as well as greater intake of cereals, roots, tubers, and milk and its derivatives, whilst females consumed more fruits and vegetables. Meat was widely consumed, although females consumed more fish and seafood than males. Eggs were hugely popular foods, in contrast to legumes. Pasta was a dietary staple in females. Visible fats and luncheon meats were consumed little. IAS values reflected the “need to change”, whilst physical activity engagement was found to be low. Conclusions: The nutritional status of the present study population was characterized by a tendency toward increasing overweight, particularly amongst females, with the predominance of class 1 obesity, low physical activity, and a healthy eating index requiring change toward more healthy habits.

## 1. Introduction

Defining dietary habits, engagement in physical activity for health or recreational reasons and nutritional status in older adult populations constitutes an important source of knowledge for the design and evaluation of social policies or programs targeting nutritional care [1].

The aforementioned situation takes on greater importance in Latin America, specifically in Ecuador, whose demographic is characterized by low fertility rates, increased life expectancy at birth, continuously reduced mortality, and increased longevity [2,3]. As a consequence, there is an emerging group of adults aged 65 years and above who are considered to be older adults [4] and are going through a progressive aging process which prevents them from being socially productive, whilst increasing their need for hospital care. 

According to Sastre Gallego [5], during the aging process, malnutrition is a common disorder amongst older adults. This is as much in the sense of deficiencies in the energy–protein balance as in the consequences of excess consumption, leading to comorbidities and chronic diseases that affect quality of life. Alongside other authors [6,7], this author highlights that no consensus exists regarding dietary or nutritional recommendations for this group of individuals, with those developed for adults in general being used to serve this population. To that discussed above, it must also be added that the simple existence of a dietary guideline does not provide any guarantee that the individuals to which it is directed will meet it. This is the case with older adults whose dietary habits are widely heterogenous [8], standing out from other populations with regard to their diversity, whilst also constituting a significant risk factor for the development of chronic diseases during aging.

Gil Gregorio et al. [7] encouraged the application of criteria previously laid out by Barrón [8] and recommended use of the NAOS (Nutrition, Physical Activity, and Obesity Prevention) strategy to promote healthy diets. At the same time, physical activity engagement is encouraged as a means to reversing trends toward greater obesity during old age and, consequently, reducing mortality associated with chronic disease [9].

In accordance with that discussed, the aim of the present work was to examine dietary habits, nutritional status, and physical activity engagement in older adults in the city of Manta, Manabí.

## 2. Materials and Methods

### 2.1. Type of Study

An observational, descriptive, and cross-sectional study conducted with older adults from Canton Manta (Manabí, Ecuador) is presented.

### 2.2. Definition of the Population and Study Sample

The population was formed by older adults from Canton Manta which, according to the National Statistics and Census Institute [10], comprises 11,943 individuals, with a slight gender difference (7%) favoring women. Probabilistic randomized sampling was performed with the sample being stratified according to sex via the equation defined by Torres et al. [11] for finite populations. The level of significance was set at 95% (α = 0.05), whilst specific error or precision was 3% (d = 0.03). A final sample of 130 participants was recruited, of which 60 men and 70 women were selected via a random numbers chart produced using registrations at Manta city parish. The research was carried out between the months of January 2021 and March 2022.

Inclusion criteria dictated that all participants must belong to an age group corresponding to older adults, be physically and cognitively sound, and be autonomous at the time of making decisions.

As exclusion criteria, individuals were not considered for inclusion if they were married couples living in the same household, individuals undergoing some type of ongoing treatment for a chronic or dietary/nutritional disorder, or individuals with dietary allergies or intolerances which could modify the consumption of foodstuffs.

### 2.3. Instruments and Methods

The below-described methods and instruments were used to gather data on the main study variables, with a focus on nutritional status, dietary habits, and physical activity engagement.

In order to determine nutritional status of participating individuals according to anthropometry, participants’ height (H, m) and weight (W, kg) were measured using the protocol stipulated by Marfell-Jones et al. [12] The weight was obtained using a Tanita InnerScanV Model e-scale: BC-545N, with an accuracy of 0.1 kg. Height was determined using a portable SECA 206 vertical stadiometer with a range of 0–220 cm and 1 mm precision. All measurements were taken during the early hours of the morning at local community facilities or in the participant’s own home. A designated family member or friend was present at all times throughout measurement. Participants wore light clothing and were instructed to empty their bladder prior to measurement. From these measures, body mass index was calculated (BMI, kg/m^2^). Nutritional status was then classified according to that proposed by the Spanish Society of Parenteral and Enteral Nutrition (SENPE) [13].

Dietary habits and the frequency of the consumption of foodstuffs were determined following administration of a food frequency questionnaire. The consumption questionnaire presented by Aguirre et al. [14] was adapted to target the study population, in line with methodological considerations defined by Muñoz-Cano et al. [15] in Tabasco (Mexico) and Hernández Gallardo et al. [16] with adolescents and young adults from Ecuador. This questionnaire comprised 11 categories pertaining to the diet. Each category included a list of foodstuffs, of which some were staples in the region of Manabí and Ecuador [17]. These categories were later organized into three sections, according to recommendations included in the Strategy for Nutrition, Physical Activity, and Obesity Prevention (NAOS Pyramid), [7] and later used to evaluate the quality of the Spanish diet (Table 1) [18].

In addition, temporal patterns around feeding were examined in the study population. These were based around three main timepoints: breakfast (from the first hour of the morning to 10:00 a.m.), lunchtime (from 12:00 to 3:00 p.m.), and dinner (from 7:00 p.m. onward). Alongside the timepoints described above, regular consumption of foods during mid-morning and late afternoon were added, as was sporadic intake outside of the aforementioned times. Such intake was denominated as snacks/picking for their quantification in the present work. Together with this, physical activity engagement for health or recreational reasons was determined, in addition to the frequency of weekly engagement by participants. All of these data were obtained through self-report via a direct interview with participants at the same time as performing anthropometric measurements. 

For the interpretation of consumption frequency and calculation of the healthy eating index (IAS), the scoring approach described by Norte Navarro and Ortiz Moncada [18] was used. Ratings were produced that ranged from “daily consumption” to “never/almost never” and corresponded to a scoring range of 0 to 10 as presented by the above authors (Table 1), in accordance with healthy intake and physical activity guidelines for elderly individuals in Spain outlined by Gil Gregorio et al. [7]. IAS values were obtained by adding together the individual values recorded for each participant, with a maximum possible score of 110. Resultant scores were distributed as follows: scores ≥80 were classified as “healthy”; scores between 80 and 50 were classified as “requires change”; scores >50 were considered to “contribute little to good health”.

### 2.4. Statistical Analysis

Using the obtained data, a database was created in the statistical program SPSS version 23. Means (MD) and standard deviations (SD) were calculated, appearing throughout the present work as MD ± SD. Further, simple frequencies and percentages were calculated for nominal variables. Normality of the data was checked via the Kolmogorov–Smirnov test, revealing a non-normal data distribution in the case of anthropometric variables. As a result, hypothesis testing was performed using the U-Mann–Whitney test (Z) (α = 0.05). The Pearson test (r) was used to examine correlations between continuous variables, and r^2^ coefficients were calculated. Meanwhile, Spearman (Rho) correlations were performed for categorical variables. The Z test (α = 0.05) was employed to compare proportions, using the Bonferroni correction to adjust the p-value. 

### 2.5. Ethical Considerations

With regard to ethical considerations, it is important to mention that participants provided written informed consent, in accordance with the bioethical principles laid out in the Declaration of Helsinki and the ethical protocols for research outlined by “Eloy Alfaro” Secular University of Manabí (Ecuador). The present study conforms to a “Dietary Habits, Physical Activity and Health in Populational Groups Pertaining to the Coastal Region of the Republic of Ecuador” project report (Resolution No. 0178-2017-FCCEE-BMM. ULEAM), included within the “Health, Physical Culture, and Social Services” line of research being ran at the Eloy Alfaro Secular University of Manabí.

## 3. Results

Participants in the present study had an average age of 71.10 ± 4.34 years in the case of males and 72.06 ± 4.88 in the case of females. Males weighed more and were taller than females (Table 2), with this difference being statistically significant (Z_P_ = −2.96, *p* = 0.000; Z_T_ = −3.96, *p* = 0.003). Bivariate correlations (r) among weight (W), height (H), and age (A) revealed no significant association between the first two measures (r = 0.763; *p*-value 0.06) or between H and A (r = 0.163; *p*-value = 0.63). Nonetheless, outcomes pertaining to weight and age differed, producing a significant correlation (r = 0.92 and *p*-value = 0.000) and a determination coefficient (r^2^) of 85%. With regard to BMI, values were higher amongst women than amongst males, with females being classified accordingly into the category pertaining to class 1 obesity and males being classified as overweight. 

Taking BMI as a main indicator, participants’ nutritional status was negatively affected by the excess body mass seen in the study sample. Nonetheless, in terms of nutritional categories, most normal-weight individuals were men (Table 2), whilst most individuals found in the groups pertaining to overweight were women. A total of 83.07% of older adults exhibited at-risk nutrition due to their excess body mass, with this being especially the case amongst women. Furthermore, a highly significant correlation (r) was found between weight and height, with these also being significantly associated with BMI. Nevertheless, when considering the association of these variables with age, no significant relationship was produced with height (r = 0.163; *p* = 0.063), although it was with weight (r = −0.292; *p* = 0.001) and BMI (r = 0.488; *p* = 0.000).

With regard to dietary habits, it can be seen in Table 3 that vegetables corresponded to the food most commonly consumed every day (84.62%). Fruits were less often consumed, although this situation does not seem to be of major concern given that the sum of individuals reporting consuming fruit on a daily basis and at least three times a week was the same as the proportion reporting daily vegetable consumption. These dietary categories were largely represented, and reporting corresponded to frequencies that demonstrated high intake over the course of a week.

With regard to protein, Table 4, pertaining to foods of weekly consumption, presents proteins that are both animal- and plant-based. With regard to meats, perfect agreement was found between the number of individuals consuming this food on a daily basis (38.46%) and those who reported only consuming it once or twice a week. Indeed, 95% of men reported consuming meat on a weekly basis compared to 90% of women, with more women consuming meat on a daily basis. A different situation emerged in relation to the consumption of fish and seafood, with this generally not being included on the menu on a day-to-day basis. In fact, 43.85% of respondents stated consuming these foods three or more times a week, whilst 48.46% did so once or twice a week, with women reporting higher intake generally. In the case of eggs, this was a widely consumed food, with daily intake being reported by 56.92% of respondents, mostly women, whilst 14.62% of all participants reported consuming eggs three or more times a week, of which only 13.07% were men. 

In the overall sample, the consumption of legumes as a source of plant-based protein was reported by less than 31% to occur on a daily basis, once or twice a week, and three or more times a week, with no statistical differences in the frequency of reporting these three categories (*X*^2^ = 15.12; *p* = 0.052). Furthermore, 14.62% of respondents stated that they consumed legumes less often than once a week. Women stood out as reporting more regular consumption of this food than men. 

It can be observed in Table 5 that pasta was a staple of the regular diet throughout the week. In this sense, women reported regular intake, whilst most men consumed this food once or twice a week. Sweet foods, such as pastries, were consumed by 92.3% of present elderly participants, with a preference emerging amongst men, despite no statistically significant differences according to sex (*X*^2^ = 39.739; *p* = 0.000). Only 7% of study participants reported abstaining totally from this food. Next, visible fats and luncheon meats are found, ordered according to the frequency of their consumption. With regard to visible fats, men were found to have a higher intake than women, whilst women exceeded the intake of their male counterparts with regard to luncheon meats.

With regard to feeding times (Table 6), the most common dietary pattern adhered to breakfast, lunch, and dinner, particularly amongst women, followed by a dietary pattern made up of consuming five or more smaller meals a day, a pattern that predominated amongst men. Even when the overall sum of the two reported types of dietary patterns was considered, it is clear that no differences existed with regard to the proportional consumption of different foods, as manifested via the confidence interval produced for α = 0.05 following comparison testing of the respective proportions (Z). This being said, men showed a behavioral tendency toward a feeding pattern comprising a higher number of feeding times or meals. 

Limited engagement in physical activity was observed in the older adults in the present study (Table 7). A total of 30% stated going for walks once a week to improve their health, whilst a similar percentage of respondents stated exercising between two and three times a week, adding to their walks more varied activities such as ball games and short runs.

Table 7 presents the classification of study participants according to the healthy eating index. Here, it can be seen that the overriding status within the population was “requires change”, with no statistically significant differences being found between men and women, as manifested through the confidence intervals for α = 0.05.

The consumption of water, fizzy drinks, and natural juices showed a large degree of variation within the studied population, although a strong preference was seen for the consumption of water over the other types of beverages. Thus, 77% of participants drank more than four glasses a day, pertaining to 80% in women and 73% in men. Only 7.7% of the overall sample drank one glass of water a day, pertaining to 2.3% of men and 5.4% of women. These individuals did not drink carbonated drinks or natural juices pointing to a clear manifestation of hypodipsia. In contrast to the consumption patterns reported for water, it was established that 30.8% of the overall sample abstained completely from fizzy drinks, and 38.46% consumed them infrequently (less than once a week). Natural juices were consumed instead of fizzy drinks and as an adjunct to water. Indeed, 61.5% of all respondents examined in the present work consumed this type of beverage on a daily basis, with 46.2% of men and 53.8% of women reporting drinking juice.

## 4. Discussion

Findings of the present study revealed that older adults from Canton in Manta show a general tendency toward an increase in body weight throughout aging, with this trend being stronger in women than in men and with a greater prevalence of class 1 obesity in both sexes. Furthermore, the difference seen in the number of men relative to women classed as being of normal weight, alongside the statistically significant correlation found between age and weight, but not with height, supports the idea the aging acts as an obesogenic factor in women and, to a lesser extent, in men. However, it would be too simplistic to consider increasing age as a determinant of overweight. Indeed, a number of authors have argued that individual differences are determined by genetic factors and add to this the finding that environmental factors account for between 60% and 70% of weight gain [19,20]. Such a situation suggests that study participants experience a decrease in their daily energy expenditure or an increase in their dietary consumption. This reality has already been discussed by Lam et al. [21] and Valdés Badilla et al. [22]. 

In this sense, the nutritional situation described above differs from that put forward by Barrón et al. [8] in relation to older adults from Chile. The latter population exceeded that from Ecuador examined in the present study in terms of both body weight and height, although the former sample tended toward normal weight. On the other hand, when contrasting the older adults from Manta recruited to the present study with participants of another similar study carried out in Guayaquil, another coastal region of Ecuador, by Álvarez Córdova et al. [23], the present sample again stands out for its lower weight and height. However, in this case, similar rates of overweight status were seen, above all in women. 

It should be mentioned that the excess weight found within the study population was discussed by Zayas et al. [24] and Michalakis et al. [25] as being a hallmark of the third age, with this same state also being mentioned by Stevens et al. [26] and Sezginsov et al. [27] as an adaptive factor for achieving satisfactory and healthy aging. These authors consider that individuals who are overweight when they begin this stage of the lifecycle tend to remain free of morbidities and disabilities for longer throughout the aging process. This same condition was previously observed by Albert Cuñat et al. [28] in Mexican elderly through the observation that individuals weighing 15% less than their ideal weight had higher mortality. Furthermore, Speakman [29,30] added that the accumulation of fat provides a metabolic reserve that helps tackle disease and, particularly, the need to survive pathogen-induced periods of anorexia.

The aforementioned overweight issue, regardless of its adaptive repercussions, could be due to a chronically positive nutritional energy balance. This leads to an increase in fat mass and a decrease in muscle mass due to the reduced motor activation seen in these individuals who require tailored nutritional recommendations [31], as opposed to the ‘one size fits all’ approach taken by extending the recommendations established for younger age groups. Such a tailored approach would include physical activities as a means to regulate calorie intake and body weight [32]. This would contribute to the adoption of a healthy lifestyle and support satisfactory aging.

With regard to dietary habits, a high intake of dietary fiber stood out through a wide array of plant-based foods, of which vegetables represented the main staple foodstuff, followed by roots, tubers, cereals, and fruits. All of these foods contribute to maintaining healthy bowel movements and preventing constipation [24], in addition to providing an important source of micronutrients [33]. Preferences differed according to sex, with men opting more for cereals, roots, and tubers, whilst women showed a preference for vegetables, green vegetables, and fruits. This preference toward plant-based foods denotes that their dietary approach has some superior qualities to that of young people from the same area [16]. This same finding was also reported by Candía et al. [34] in Chilean older adults when compared to young people of the same nationality. 

Consumption of dairy products in the present study population contradicts that previously reported by Barrera et al. [35], who uncovered a limited intake in a population of the Pacific Coast region. It could be concluded that present findings reveal that men consume dairy products every day whilst women do so at least three times a week. In this way and as stated by Fernández et al. [36], intake promotes benefits for the cardiovascular and cognitive systems, as well as for bones, muscles, and joints. 

Consumption of animal-based foods as a source of protein was characterized, in the present study, by wide variation and biological quality. A varied intake of meat, fish, and seafood was seen, setting the present sample apart from others in continental regions who, according to Moos et al. [37], tend more toward red meats and abstain from fish and other sea-sourced foods. Furthermore, eggs are widely used in different culinary forms, appearing as a daily staple in different forms, largely due to reports given by women. High consumption of this food reflects dietary habits inherited from the Western world [37,38].

Legumes, as sources of protein and dietary fiber [39], were found to be consumed in limited amounts by the study sample, with consumption even being lower than that reported for inhabitants of Colombia and Chile according to a study conducted by Guerrero et al. [40]. This finding shows that older adults examined in the present work prefer animal-based foodstuffs as a source of protein over plant-based foods. Biological quality is ensured, and monotonous intake avoided by consuming different specific foods. Such monotony is conceptualized by the Pan-American Health Organization as the anorexia of aging [41], who also declared it to be one of the main causes of malnutrition within aging populations. 

The consumption of foods in the present sample classified as occasionally consumed foods pointed to an at-risk nutritional status. This was especially due to the consumption of foods made with refined flours, luncheon meats, and visible fats. All of these foods are appealing not just because of their pleasant taste but also because they are easy and quick to digest [1], are capable of stimulating the palate [28], and can be used as a main dish or as an accompaniment. In the case of the latter, they act to facilitate the introduction of other foods. 

In the present elderly sample, a dietary pattern characterized by three feeding or meal times predominated, namely, breakfast, lunch, and snack/dinner. This coincides with findings reported with Chilean older adults [8], although a trend was seen toward increasing the number of feeding or meal times toward five times a day. Such a dietary pattern was accommodated by adding a meal in the early evening hours and an additional evening meal. Only 10% of individuals reporting such patterns were found to be overweight, whilst 60% of those reporting the former pattern were overweight. This finding is supported by other authors [25,42] and suggests that fewer meal times may correspond to a greater overall food consumption.

Findings obtained in relation to the healthy feeding index (IAS) reflect the existence of inadequate feeding habits and the need to adopt changes to acquire healthier nutrition. This is not an isolated issue as shown by Hernández Galiot and Goñi Cambrodón [43], who previously reported the same situation in elderly individuals from Spain and the US. In the case of the present research, only a small proportion of participants were found to adhere to the dietary and physical activity guidelines laid out in the NAOS reference pyramid. 

Engagement in physical activity for health or recreation was found to be limited in the present sample, similarly to that reported by Álvarez Córdova et al. [23] in older adults from Guayaquil. It should also be highlighted that all of the elderly individuals in the present study who abstained from physical activity engagement were also found to be overweight, a finding also reported by Rodríguez-Rodríguez et al. [19] in a Spanish population.

### Limitations to the Study

The stratification of the sampling, according to sex, determined that the group of women was superior to that of men, influencing the value of the total group. The nature of the cross-sectional descriptive observational study limits the identification of cause–effect relationships.

## 5. Conclusions

The nutritional status of the examined population showed a tendency toward increasing weight alongside increasing age, with class 1 obesity predominating as a clear indication of a chronic positive nutrition/energy balance.

The dietary habits of the elderly adults in the present study were characterized by three main meal times, the consumption of diverse plant-based foods and varied protein sources, a trend toward the consumption of refined flour, and a pattern comprising drinking four glasses of water a day, complemented with natural juices and the avoidance of carbonated drinks. Overall, such a profile helps to avoid dietary monotony or the anorexia of aging; however, in consideration of the healthy eating index, changes are required to achieve a healthy dietary status.

Physical activity via sport and recreation was practiced by a limited number of the present sample, pointing to a characteristic trait of this aging group.

The Council the Barrios de Manta and the participants are thanked for their availability and collaboration with the study.

## Figures and Tables

**Table 1:** Recommendations for the frequency of consumption of determined foods according to dietary groups, dietary components, and score used to determine the healthy eating index (IAS).

Expected Consumption Frequency	Food Group	Description of Foods	Criteria for the Scoring of IAS				
			10	7.5	5	2.5	0
1. Daily intake	1. Cereals and derivatives	Rice, corn-based foods, bread or cracker, noodles, oats, amaranth, quinoa	Daily intake	3 or more times a week	1 or 2 times a week	Less than 1 time a week	Never or almost never
	2. Green vegetables and vegetables	Carrots, spinach, lettuce, cauliflower, peppers, tomatoes, broccoli, celery, chard, other					
	3. Roots, tubers and plantain.	Potatoes, green plantain, ripe plantain, yuca, camote, pumpkin, other					
	4. Fresh fruit and natural juices without added sweeteners	Apple, orange, tree tomato, watermelon, melon, pineapple, pear, bananas, other					
	5. Milk and derivatives	Milk, yogurt, cheese, cottage cheese, other					
2. Weekly intake	6. Meat, seafood, fish and eggs	Meats: poultry, pork, beef, fowl, other	3 or more times a week	1 or 2 times a week	Less than 1 time a week	Daily intake	Never or almost never
		Seafood: shrimp, clams, oysters, other.					
		Fish: deep-water and/or saltwater fish					
		Eggs: hens, quail, others from poultry.					
	7. Legumes	Black beans, green beans, peas, lupin, chickpeas, white beans, lentils, soy and similar					
3. Occasional intake	8. Pasta	Spaghetti, tagliatelle, cannelloni, lasagna, pizza, other	Never or almost never	Less than 1 time a week	1 or 2 times a week	3 or more times a week	Daily intake
	9. Sweets	Made from wheat flour or glazed fruit, white bread, biscuits, added sugar, jams, ice cream, pastries, cookies, sugary natural juices, fizzy drinks.					
	10. Luncheon meats	Sliced ham, chorizo, sausage, hot dog, hamburger, cured meat, other					
	11. Visible fats	Butter, margarine, mayonnaise, solid cooking fats, cream cheese such as Philadelphia, other.					

**Table 2:** Mean and standard deviation pertaining to weight, height, and BMI.

Sex	Weight (W, kg)	Height (H, m)	BMI	Normal Weight		Overweight		Class 1 Obesity		Class 2 Obesity	
				f(x)	%	f(x)	%	f(x)	%	f(x)	%
**Men (n = 60)**	62.53 ± 9.18	1.56 ± 0.06	29.28 ± 3.24	20	33.33	12	20	25	41.67	3	5
**Women (n = 70)**	57.4 ± 4.13	1.51 ± 0.06	31.30 ± 2.45	2	2.86	26	37.14	35	50	7	10
**Total (n = 130)**	59.76 ± 7.36	1.53 ± 0.07	30.37 ± 3.01	22	16.92	38	29.23	60	46.15	10	7.69

**Table 3:** Consumption frequency of food groups according to food intake. Classification in accordance with dietary recommendations for older adults. Daily consumed foods.

Sex	Frequency	Cereals		Vegetables			Roots/Tubers/Plantain			Fruit				Milk/Derivatives	
		Daily	>2/ Week	Daily	>2/ Week	1 or 2/Week	Daily	>2/ Week	1 or 2/Week	Daily	>2/ Week	1 or 2/Week	<1/ Week	Daily	>2/ Week
**Men** **n = 60**	f(x)	48	12	47	10	3	48	6	6	38	16	3	3	48	12
	%	80	20	78.33	16.67	5	80	10	10	63.33	26.67	5	5	80	20
**Women** **n = 70**	f(x)	42	28	63	0	7	42	14	14	42	14	7	7	42	28
	%	60	40	90	0	10	60	20	20	60	20	10	10	60	40
**Total** **n = 130**	f(x)	90	40	110	10	10	90	20	20	80	30	10	10	90	40
	%	69.23	30.77	84.62	7.69	7.69	69.23	15.38	15.38	61.54	23.08	7.69	7.69	69.23	30.77

**Table 4:** Consumption frequency of food groups according to food intake. Classification in accordance with dietary recommendations for older adults. Weekly consumed foods.

Sex	Frequency	Meat				Seafood/Fish			Eggs			Legumes			
		Daily	>2/ Week	1 or 2/Week	<1/ Week	Daily	>2/ Week	1 or 2/Week	Daily	>2/ Week	1 or 2/Week	Daily	>2/ Week	1 or 2/Week	<1/ Week
**Men** **n = 60**	f(x)	22	13	22	3	3	22	35	20	17	23	15	19	23	3
	%	36.67	21.67	36.67	5	5	36.67	58.33	33.33	28.33	38.33	25	31.67	38.33	5
**Women** **n = 70**	f(x)	28	7	28	7	7	35	28	54	2	14	23	21	10	16
	%	40	10	40	10	10	50	40	77.14	2.86	20	32.86	30	14.28	22.86
**Total** **n = 130**	f(x)	50	20	50	10	10	57	63	74	19	37	38	40	33	19
	%	38.46	15.38	38.46	7.69	7.69	43.85	48.46	56.92	14.62	28.46	29.23	30.77	25.38	14.62

**Table 5:** Consumption frequency of food groups according to food intake. Classification in accordance with dietary recommendations for older adults. Occasionally consumed foods.

Sex	Frequency	Pasta			Sweets/Flour					Luncheon Meats					Visible Fat				
		Daily	>2/ Week	1 or 2/Week	Daily	>2/ Week	1 or 2/Week	<1/ Week	Never	Daily	>2/ Week	1 or 2/Week	<1/ Week	Never	Daily	>2/ Week	1 or 2/Week	<1/Week	Never
**Men** **n = 60**	f(x)	9	4	47	3	20	28	6	3	12	16	8	11	13	3	19	3	26	9
	%	15	6.67	78.33	5	33.33	46.67	10	5	20	26.67	13.33	18.33	21.67	5	31.66	5	43.33	15
**Women** **n = 70**	f(x)	30	7	33	35	14	14	0	7	21	7	0	21	21	7	21	7	14	21
	%	42.86	10	47.14	50	20	20	0	10	30	10	0	30	30	10	30	10	20	30
**Total** **n = 130**	f(x)	39	11	80	38	34	42	6	10	33	23	8	32	34	10	40	10	40	30
	%	30	8.46	61.54	29.23	26.15	32.31	4.62	7.69	25.38	17.69	6.15	24.62	26.16	7.69	30.77	7.69	30.77	23.08

**Table 6:** Distribution of participating older adults according to feeding times.

Sex	Frequency	Breakfast, Lunch, Dinner (Snack)	Lunch and Grazes on Something Later	Lunch, Grazes and Dinner (Snack)	>4 Meals	Total
Men	f(x)	31	3	3	23	60
	%	51.67	5	5	38.33	100
Women	f(x)	49	7	7	7	70
	%	70	10	10	10	100
Total	f(x)	80	10	10	30	130
	%	61.54	7.69	7.69	23.08	100

**Table 7:** Physical activities engaged in for recreation of health, frequency of engagement, and healthy eating index.

Sex	Freq.	Physical Activity					Frequency of Physical Activity				IAS	
		Walking for More Than 30 min	Running	Aerobic Exercise	Varied Exercise	None	1/ Week	2 or 3/ Week	>3/ Week	0	Healthy	Requires Change
**Men**	f(x)	15	10	3	13	19	18	19	3	20	31	29
	%	25	16.67	5	21.67	31.67	30	31.67	5	33.33	51.67	48.33
**Women**	f(x)	35	0	7	7	21	21	21	7	21	20	50
	%	50	0	10	10	30	30	30	10	30	28.57	71.43
**Total**	f(x)	50	10	10	20	40	39	40	10	41	51	79
	%	38.46	7.70	7.70	15.38	30.77	30	30.77	7.70	31.54	39.23	60.77

## Data Availability

Data are not publicly available due to the fact that the study will be conducted at other centers.
